# Endometriosis Grade 4 in In Vitro Fertilisation and Its Management: A Case Report

**DOI:** 10.7759/cureus.47455

**Published:** 2023-10-22

**Authors:** Abhijeet Raj, Kshiti P Deshpande, Neema Acharya

**Affiliations:** 1 Department of Medicine, Jawaharlal Nehru Medical College, Datta Meghe Institute of Higher Education and Research, Wardha, IND; 2 Department of Obstetrics and Gynaecology, Jawaharlal Nehru Medical College, Datta Meghe Institute of Higher Education and Research, Wardha, IND

**Keywords:** bladder endometriosis, severe endometriosis, endometriosis staging, endometriosis prevention, endometriosis surgery, endometriosis diagnosis

## Abstract

Endometriosis is a condition in which tissue that lines the uterus grows outside the uterus. Patients with endometriosis often experience pelvic pain with menstrual periods and sometimes also infertility. Sometimes it is mistaken for pelvic inflammatory disease or ovarian cysts. This condition is a contraindication for pregnancy. This is a case report of a 37-year-old female who came to in vitro fertilisation (IVF) with a history of infertility of 14 years and was diagnosed with Grade 4 endometriosis on diagnostic ultrasonography-guided hysterolaparoscopy. After many trials of intrauterine insemination she was advised to go for IVF, and that not by obtaining her own ovum. The donor’s egg and male partner's sperm were used for the IVF. The fertilised zygote was then implanted in the patient's uterus.

## Introduction

A condition known as endometriosis causes tissue that resembles the uterine lining to protrude outside of the uterus. The endometrium is the term for the lining of the uterus. Endometriosis manifests as the growth of endometrial-like tissue on the ovaries, intestines, and tissues lining the pelvis. While it's uncommon, it's possible for endometrial-like tissue to spread outside of the pelvic region. Endometrial implants are tissues that are growing outside of the uterus that mimic endometrium.

The hormonal changes associated with the menstrual cycle have an impact on the displaced endometrial-like tissue, which causes discomfort and inflammation there. This implies that the tissue will swell, harden, and degrade. The tissue that has deteriorated over time becomes trapped in the pelvis because it has nowhere else to go. The tissue that is trapped in the pelvis can lead to irritation, scarring, adhesions (where tissue binds the pelvic organs together), excruciating pain during the menstrual cycle, and fertility issues.

There are four phases or forms of endometriosis. Any of the following are possible: minimal, mild, moderate and severe. The stage of the illness is determined by a variety of circumstances. These variables may include the placement, quantity, dimensions, implantation length and depth. The fallopian tubes, the uterus's outer surface, the ovaries, and the ligaments that surround the uterus are where endometriosis lesions are most usually found. Endometriosis lesions come in a variety of sizes and frequently appear as nodules or cysts [1]. This ongoing inflammatory process increases inflammatory mediators including prostaglandins, which cause collagen degradation and cervical ripening as well as tissue malfunction. These outcomes may account for the increased risk of preterm labour and vascular rupture, respectively [2].

Each score was classified as 1 to 5, 6 to 15, 16 to 40, and more than 40. The stages were described as minimum, mild, moderate, and severe. The updated categorisation left out tubal endometriosis and divided endometriosis lesions into superficial and deep lesions according to the Revised American Society For Reproductive Medicine classification [3].

## Case presentation

A 37-year-old female patient presented with the chief complaint of infertility for 13 years. The male partner of 41 years, both married for 14 years, tried to conceive but failed every time. She was advised intrauterine insemination (IUI). This was done 3 times but it failed each time IUI was done. The ovaries of the patient were in the pouch of Douglas in the midline “Kissing Ovaries”. The distorted tubo-ovarian anatomic relationship which caused IUI failure is suggestive of poor ovarian reserve. Hence, oocytes were not retrieved despite controlled ovarian stimulation with gonadotropins. Hysteroscopy was done through a vaginoscopic approach on which the cervix and endocervical canal were normal, the uterus was anteverted, the uterine cavity was normal, and b/l ostia was seen as normal. 

Later digital hysterosalpingography study was done on which mild dilatation of the infundibulum and fimbrial end of both sides fallopian tube was seen. There was no e/o free peritoneal spill suggestive of cornual blockage. Then she was advised ultrasonography guided hysterolaparoscopy which revealed the female had septum at the fundus bowel, with the omentum completely covering the uterus, the sigmoid colon densely adherent to the fundus and the ovaries in the pouch of Douglas. Later laparotomy was done to see the exact organs.

The drugs given to the patient after diagnostic hysteroscopy were as shown in Table 1.

**Table 1:** Drugs prescribed OD: Once daily, TDS: Thrice daily

Drugs prescribed
Cephalosporin Inj 1 gm IV 12 hrly OD
Pantoprazole 40 mg IV 12 hrly
Inj Meteronidazole 100 CC IV Drip IV 12 hrly
Inj Tramadol Hydrochloride 2 CC IV IN Drip IV 12 hrly
Zonac Supp/R TDS

Her hormonal assay reports showed the following: follicular stimulating hormone (FSH), oestradiol (E2), luteinizing hormone (LH), anti-Mullerian hormone (AMH), and body mass index (BMI). The hormonal assay reports showed that oestrogen was low which was a risk for pregnancy (Table 2). All other parameters were within normal limits.

**Table 2:** Hormonal assay reports FSH: Follicular stimulating hormone, E2: Oestradiol, LH: Luteinizing hormone, AMH: Anti-Mullerian hormone, BMI: Body mass index

Investigation	Observed value	Unit	Biological reference range
FSH	7.13	m IU/ml	1.5 – 12.4
E2 (Oestradiol)	11.72	pg /ml	30 - 400
LH	8.32	M IU/ml	5 - 25
AMH	0.365	ng/ml	0.02-11
BMI	>30	-	18.5 – 24.9

The thyroid profile, that is triiodothyronine (T3), thyroxine (T4), and thyroid stimulating hormone (TSH) were within normal limits (Table 3).

**Table 3:** Thyroid profile T3: Triiodothyronine, T4: Thyroxine, TSH: Thyroid stimulating hormone

Investigation	Observed value	Unit	Biological reference range
T3	1.3	ng/ml	0.97-1.69
T4	11.8	Microgram/dl	5.5-11.0
TSH	1.60	Micro IU/ml	0.46-4.68

**Figure 1:**
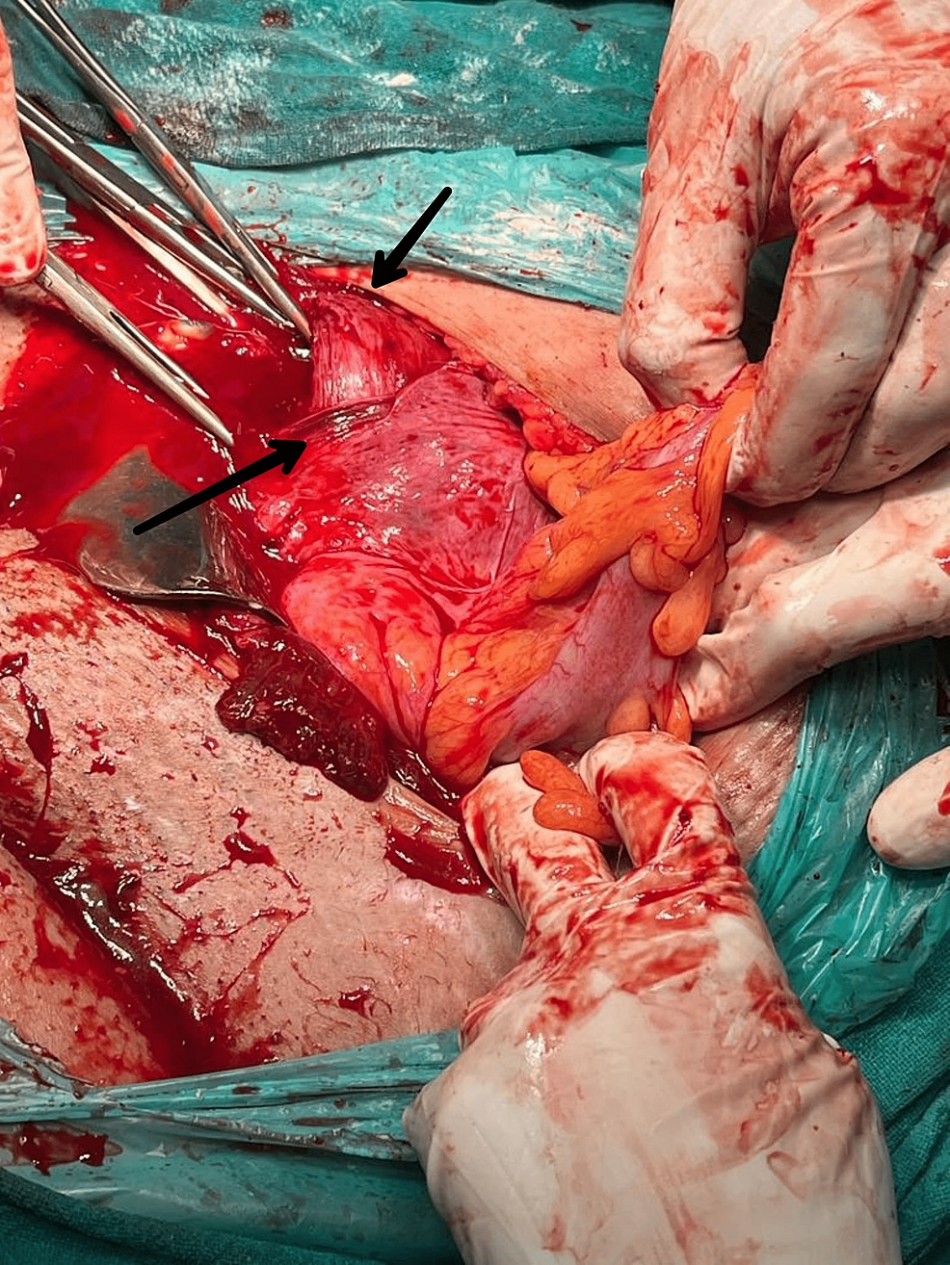
Intraoperative image

There were severe adhesions as shown in the intraoperative image from a laparoscopy procedure (Figure 1). Therefore, donor oocytes were chosen for in vitro fertilisation. The husband’s semen analysis parameters were within normal limits. So, the IVF was done with donor locustes and the male partner's sperm and implanted in the uterus of the female partner.

## Discussion

The surgical finding of endometrial tissue outside of the uterine cavity is the conventional anatomic diagnosis of endometriosis; nevertheless, this has not been found to be adequate to explain the disease's natural course, the full range of its clinical features, the frequent recurrence of its symptoms, the underlying molecular pathophysiology, or its responsiveness to the currently available management options [4]. Anovulation, aberrant follicular development with stunted follicle size, decreased levels of circulating E2 during the preovulatory phase, and luteinizing hormone disruption are all symptoms of endometriosis (LH). Surge patterns, premenstrual spotting, the syndrome of a luteinized unruptured follicle, galactorrhoea, and hyperprolactinemia, an analysis just completed says [5]. An increasing body of research shows that immunological and hormonal processes promote inflammation and support the survival of endometriosis. This has two primary signs of the illness, pain and infertility. The endocrine and inflammatory processes involved in the pathophysiology of the illness are pharmacologically impacted by new medications on the market. The pathogenesis of endometriosis will be studied in new ways as a result of this [6].

Endometriosis shares some characteristics with malignancies, such as invasive, oestrogen-dependent growth, recurrence, and a propensity to spread. Endometriosis, a crippling illness with signs of chronic inflammation, is mentioned in. According to estimates, 10-15% of women of reproductive age have pelvic endometriosis, making it one of the most prevalent benign gynaecological proliferations in premenopausal women. Uncertainty surrounds the biology of endometriosis. Despite being widespread, this illness is still poorly understood, and recent research has disproven the notion that symptoms and severity of the illness are related. The diagnosis of endometriosis cannot be made via a blood test. There is currently no one very effective method for treating endometriosis. The relative ineffectiveness of hormonal therapy for endometriosis has led to the clinical testing of a number of alternative experimental therapies [7]. The relative ineffectiveness of hormone treatment for endometriosis has led to the clinical testing of a number of other experimental medicines. It has been proposed that endometriosis alters the uterine environment by making the endometrium resistant to progesterone and by affecting the quality of the oocyte, which may have a negative impact on embryo development and implantation. Furthermore, studies have demonstrated that endometriosis-affected women have higher levels of systemic, peritoneal, and local inflammation [8].

Endometriosis patients temporarily believe they have a cure after their first IVF pregnancy [9]. There were higher rates of miscarriage, threatened miscarriage, preterm labour, preterm delivery, placental abruption, and caesarean section in endometriosis patients. The presence of adenomyosis was found to be significantly correlated with pregnancy-induced hypertension and preeclampsia. There was no difference in the foetal fate. Pregnancy-related hemoperitoneum was reported in one instance. Obstetric complications, like miscarriage, threatened miscarriage, preterm labour, preterm birth, and a higher caesarean section rate, are more likely to occur during pregnancy in women with endometriosis. There is no evidence that endometriosis affects foetal health [10]. Infertility is a complication of moderate to severe endometriosis, which affects the ovaries and results in adhesions that prevent tubo-ovarian motility and ovum pickup.

In this case, the patient was not ready at first to do any of the procedures; so, the patient was given a trial for IUI 3 times. After the failure the patient was counselled and further management was done. The patient did not have a peritoneal spill, so the ovum was impossible to retrieve; the ovary store could not be retrieved because there was a fimbrial block. So it was difficult to convince the patient afterwards to go for donor locustes for further pregnancy.

## Conclusions

A female came with a chief complaint of infertility and was advised intra uterine insemination which failed three times. On diagnostic hysterolaparoscopy she was diagnosed with endometriosis grade 4. Her bladder was advanced and adhered to the colon. The ovaries lay in close proximity to the pouch of Douglas. The patient after the hysteroscopic reports was kept on medication. The patient went through many such procedures but every time there was a failure.

The patient was not fit to obtain her own ovum due to severe attachments of the uterus and colon and also the blockage of fimbrial ends. Then she was treated by IVF by obtaining the donor ovum and sperm of the male partner. The fertilized ovum then was transplanted into the uterus of the patient. Endometriosis is a very severe complication for pregnancy and can lead to infertility.
